# Whole-tumor histogram analysis of apparent diffusion coefficients for predicting lymphovascular space invasion in stage IB-IIA cervical cancer

**DOI:** 10.3389/fonc.2023.1206659

**Published:** 2023-06-19

**Authors:** Jin-mei Cheng, Wei-xiao Luo, Bang-guo Tan, Jian Pan, Hai-ying Zhou, Tian-wu Chen

**Affiliations:** ^1^ Sichuan Key Laboratory of Medical Imaging, and Department of Radiology, Affiliated Hospital of North Sichuan Medical College, Nanchong, Sichuan, China; ^2^ Department of Radiology, Panzhihua Central Hospital, Panzhihua, Sichuan, China; ^3^ Department of General Practice, Taiping Town Central Health Center, Leshan, Sichuan, China

**Keywords:** cervical cancer, lymphovascular space invasion, histogram analysis, magnetic resonance imaging, apparent diffusion coefficient

## Abstract

**Objectives:**

To investigate the value of apparent diffusion coefficient (ADC) histogram analysis based on whole tumor volume for the preoperative prediction of lymphovascular space invasion (LVSI) in patients with stage IB-IIA cervical cancer.

**Methods:**

Fifty consecutive patients with stage IB-IIA cervical cancer were stratified into LVSI-positive (n = 24) and LVSI-negative (n = 26) groups according to the postoperative pathology. All patients underwent pelvic 3.0T diffusion-weighted imaging with b-values of 50 and 800 s/mm^2^ preoperatively. Whole-tumor ADC histogram analysis was performed. Differences in the clinical characteristics, conventional magnetic resonance imaging (MRI) features, and ADC histogram parameters between the two groups were analyzed. Receiver operating characteristic (ROC) analysis was used to evaluate the diagnostic performance of ADC histogram parameters in predicting LVSI.

**Results:**

ADC_max_, ADC_range_, ADC_90_, ADC_95_, and ADC_99_ were significantly lower in the LVSI-positive group than in the LVSI-negative group (all *P*-values < 0.05), whereas no significant differences were reported for the remaining ADC parameters, clinical characteristics, and conventional MRI features between the groups (all *P*-values > 0.05). For predicting LVSI in stage IB-IIA cervical cancer, a cutoff ADC_max_ of 1.75×10^−3^ mm^2^/s achieved the largest area under ROC curve (A_z_) of 0.750, followed by a cutoff ADC_range_ of 1.36×10^−3^ mm^2^/s and ADC_99_ of 1.75×10^−3^ mm^2^/s (A_z_ = 0.748 and 0.729, respectively), and the cutoff ADC_90_ and ADC_95_ achieved an A_z_ of <0.70.

**Conclusion:**

Whole-tumor ADC histogram analysis has potential value for preoperative prediction of LVSI in patients with stage IB-IIA cervical cancer. ADC_max_, ADC_range,_ and ADC_99_ are promising prediction parameters.

## 1 Introduction

Cervical cancer is the most common malignancy in women, with approximately 600,000 new cases worldwide each year, second only to breast, colorectal, and lung cancer. It is also the fourth leading cause of cancer-related deaths in women worldwide (1). Lymphovascular space invasion (LVSI), defined as the presence of one or more cancer cell clusters in the lymphatic lumen or vasculature, is an independent risk factor for postoperative recurrence and poor prognosis (2, 3). The rate of postoperative recurrence is 2.64 times higher in early cervical cancer patients with LVSI than in patients without LVSI (4). Moreover, according to the Sedlis criteria, early cervical cancer patients with no lymph node (LN) metastasis but with LVSI require further LN dissection and postoperative radiotherapy to improve survival by eradicating micro-metastases (5, 6). In addition, LVSI is an essential factor in the decision-making process for fertility-sparing treatments (7, 8). For patients with LVSI who require fertility, a wide resection margin should be performed (5). Hence, assessment of the LVSI status before surgery is of great clinical relevance in cervical cancer treatment and prognosis prediction. However, LVSI is histologically diagnosed based on postoperative microscopic examination of surgical specimens. The preoperative prediction of LVSI is extremely challenging. Thus, exploring new noninvasive methods to preoperatively evaluate the LVSI status in cervical cancer is of great importance.

As one of the most commonly applied functional magnetic resonance imaging (MRI) techniques, diffusion-weighted imaging (DWI) can noninvasively reflect the anatomical structure and functional information of biological tissues at the microscopic level by measuring the diffusion properties of water molecules. The diffusion coefficient of water in biological tissues is expressed as the apparent diffusion coefficient (ADC), which is associated with the histological grade, aggressiveness, and clinical outcome of tumors (9–11). Compared with the traditional average ADC value of the largest tumor slice, ADC histogram analysis based on the whole tumor volume reflects the overall heterogeneity of tumors by displaying the frequency distribution and variation of all voxels within the whole lesion. Recently, some studies have demonstrated that ADC histogram analysis is valuable in predicting pelvic LN metastasis, evaluating the efficacy of chemoradiotherapy, and predicting tumor recurrence in patients with cervical cancer (12–14). However, few studies have applied ADC histogram analysis to preoperatively predict the LVSI status in cervical cancer. This study investigated the value of ADC histogram analysis based on the whole tumor volume for the preoperative prediction of LVSI in patients with stage IB-IIA cervical cancer.

## 2 Materials and methods

The study protocol was approved by the institutional review board of our hospital (No. 2023ER93-1), and the requirement for informed consent was waived due to the retrospective design.

### 2.1 Study population

From April 2021 to September 2022, patients with cervical cancer diagnosed by histopathology in our hospital were consecutively collected according to the following inclusion criteria (1): staged as IB-IIA according to the 2018 International Federation of Gynecology and Obstetrics (FIGO) staging system (15); (2) radical hysterectomy and pelvic LN dissection were performed in our hospital, and LVSI status was confirmed by postoperative histopathology; (3) pelvic MRI scan was performed within 2 weeks before surgery; (4) no tumor-related treatment (e.g., cervical conization, partial excision, radiotherapy, or chemotherapy) was performed before MRI examination and surgery; and (5) complete clinical data and imaging data of the patients were available. The exclusion criteria were as follows: (1) double primary malignancies (breast cancer or ovarian teratoma), (2) DWI with poor image quality, and (3) tumors that were too small (maximum diameter < 10 mm) to measure. The exclusion criteria were as follows: (1) double primary malignancies (breast cancer or ovary teratoma); (2) DWI with poor image quality; or (3) tumors that were too small (maximum diameter < 10 mm) to measure.

Fifty consecutive patients (age range, 34-70 years; mean age, 55.1 ± 7.4 years) were enrolled. According to the postoperative pathology, the patients were divided into two groups: patients with LVSI (LVSI-positive group, n = 24) and patients without LVSI (LVSI-negative group, n = 26). Clinical and pathological data, including age, gravidity, parity, menstrual status, HPV infection status, serum squamous cell carcinoma antigen (SCC-Ag) level, FIGO stage, and histological type, were collected from our database.

### 2.2 MRI protocols

All pelvic MRI scans were performed using a 3.0T scanner (uMR 790; United Imaging Healthcare, Shanghai, China) equipped with a phased-array body coil. **Table 1** lists the imaging sequences and scanning parameters. For contrast-enhanced T_1_-weighted imaging, the contrast agent (Magnevist; Bayer AG, Leverkusen, Germany) was intravenously administrated at a dose of 0.2 mmol/kg at the rate of 2 mL/s, followed by a 20 mL saline solution flush using an automated power injector (Spectris MR Injector System; Bayer, Leverkusen, Germany). The coverage of MRI scans was from the renal hilum to the external aperture of the vagina.

**Table 1:** Scan parameters of each sequence in MRI.

Sequence	Axial T_1_WI	Axial FS T_2_WI	Sagittal FS T_2_WI	Axial CE	Sagittal CE	Axial DWI
Technique	FSE	FSE	FSE	FSE	FSE	EPI
Repetition time/echo time (ms)	4/2	3523/96	2714/105	4/2	4/2	3625/68
Slice thickness/gap (mm)	3/0	5/1.2	4/0.4	1/0	3/0	4/0.4
Matrix	330×456	352×355	240×240	208×162	224×168	144×144
Field of view (mm)	360×260	380×320	220×220	360×280	220×220	220×220
Flip angle (deg)	12	120	120	10	10	90
Number of excitation	1	2	2	1	2	2/6
b value (s/mm^2^)	–	–	–	–	–	50, 800

T_1_WI, T_1_ weighted imaging; T_2_WI, T_2_ weighted imaging; FS, fat suppression; CE, contrast enhancement; DWI, diffusion weighted imaging; FSE, fast spin-echo; EPI, echo planar imaging.

### 2.3 Image analysis

**Figure 1:**
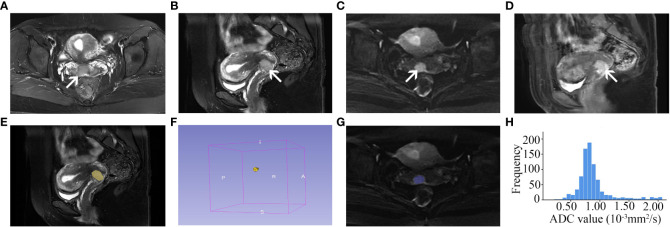
A 44-year-old cervical cancer patient with LVSI (FIGO stage IIA). Axial **(A)** and sagittal **(B)** FS T2-weighted image shows a tumor with slightly high signal intensity in the anterior lip of the cervix (arrow). The tumor is seen as high signal intensity on the axial diffusion-weighted image (**C**, arrow) and homogeneous enhancement on the contrast-enhanced image (**D**, arrow). The tumor shape is drawn on each contiguous tumor section of sagittal FS T2-weighted images to measure the tumor volume (**E**, **F**, yellow shadow). The VOI of the tumor is drawn on each contiguous tumor section of axial diffusion-weighted images (b = 800 s/mm^2^) (**G**, blue shadow), and the ADC histogram **(H)** is obtained based on the VOI. LVSI, lymphovascular space invasion; FIGO, International Federation of Obstetrics and Gynecology; FS, fat suppression; VOI, volume of interest; ADC, apparent diffusion coefficient.

All image data were analyzed in consensus by two radiologists (the first and corresponding authors with 2 and 12 years of experience in abdominal imaging, respectively) who were blinded to the clinical and pathological data of the patients. The following parameters were recorded on T_2_-weighted or delayed phase contrast-enhanced images: (1) tumor location: tumor in the anterior lip, posterior lip, or both anterior and posterior lips of cervix; (2) enhancement pattern of the tumor: homogeneous or heterogeneous enhancement; (3) maximum tumor depth/cervical radius: it was measured as a ratio of the maximum tumor depth to the cervical radius, and the measurements were classified into two grades: <1/2 or ≥1/2; and (4) tumor volume: tumor shape was manually drawn on each contiguous tumor section of T_2_-weighted images used the 3D-Slicer (version 5.0.2; https://www.slicer.org/), and then tumor volume was automatically calculated by the software (**Figure 1**).

DWI images of individual patients were uploaded to FireVoxel software (version 409; https://www.firevoxel.org/) for ADC histogram analysis. With reference to the T2-weighted and contrast-enhanced images, the volume of interest (VOI) was manually drawn along the boundary of the tumor on each contiguous tumor section of the diffusion-weighted images (b = 800 s/mm^2^). ADC histograms were obtained from the VOI (**Figure 1**). The following parameters were automatically calculated by the software: (1) ADC_mean_, the average ADC value of all voxels within the VOI; (2) ADC_min_, the minimum ADC value within the VOI; (3) ADC_max_, the maximum ADC value within the VOI; (4) ADC_n_, the point at which n% of the voxel values formed the histogram are found to the left, including the 10th, 25th, 50th, 75th, 90th, 95th, and 99th percentiles of ADC; (5) ADC_range_, the difference between ADC_max_ and ADC_min_; (6) ADC_stdev_, the standard deviation of the ADC values within the VOI; (7) ADC_coefofvar_, the ratio of ADC_stdev_ to ADC_mean_, which describes the relative dispersion degree of the ADC values; (8) ADC_skewness_, the asymmetry of the ADC value distribution around its mean. A positive skew represents most of the values to the left of the mean ADC and an asymmetric tail toward higher ADC values, whereas a negative skew indicates most of the values to the right of the mean ADC and an asymmetric tail toward lower ADC values; (9) ADC_kurtosis_, the degree of peakedness of the ADC value, which represents the concentration of ADC values around the mean and reflects the peak of the distribution; and (10) ADC_entropy_, a statistical parameter of irregularity in histograms, which reflects the disorder of the ADC value distribution.

To test the intraobserver concordance, the aforementioned measurements were repeated 30 days after the first measurement by the previous two reviewers working in consensus.

### 2.4 Histopathological analysis

All patients underwent radical hysterectomy and pelvic LN dissection at our hospital. Postoperative tumor specimens were embedded in paraffin and stained with hematoxylin and eosin. The presence or absence of LVSI was also assessed. LVSI was defined as the presence of one or more cancer cell clusters in a space lined with endothelial cells outside the tumor border.

### 2.5 Statistical analysis

Statistical analyses were performed using SPSS (version 26.0; IBM Corp., Armonk, NY, USA). *P*-value < 0.05 was considered as a statistically significant difference. The intraobserver concordance of tumor volume and ADC measurements was evaluated using the intraclass correlation coefficient (ICC). ICC values between 0.61 and 0.80 and >0.80 are indicative of good and excellent concordance, respectively; otherwise, the concordance is considered unsatisfactory. If the concordance was good, the first measurements obtained by the radiologists working in consensus were used as the final values for the subsequent analysis. Otherwise, the average of the two measurements was used as the final analysis value.

Continuous variables with a normal distribution are presented as mean ± standard deviation and were compared using the independent samples *t*-test, whereas non-normal distribution variables are presented as median (lower quartile, upper quartile) and were compared using the Mann–Whitney U test. The chi-square test was performed to compare qualitative variables between the LVSI-positive and LVSI-negative groups. The performance of ADC histogram parameters in predicting LVSI was evaluated using receiver operating characteristic (ROC) analysis.

## 3 Results

### 3.1 Patient characteristics and conventional MR imaging features: LVSI-positive vs. LVSI-negative group

**Table 2** presents the patient characteristics and conventional MRI features of the LVSI-positive and LVSI-negative groups. No significant differences were found in age, gravidity, parity, menstrual status, HPV infection status, SCC-Ag, FIGO stage, histological type, tumor location, tumor enhancement pattern, tumor volume, or maximum tumor depth/cervical radius between the groups (all *P*-values > 0.05).

**Table 2:** Comparisons of clinicopathologic characteristics and conventional imaging features between cervical cancer patients with and without LVSI.

Parameters	LVSI-positive group (*n*=24)	LVSI-negative group (*n*=26)	*P* value
Age (year)	53.1 ± 7.1	57.0 ± 7.3	0.065
Gravidity (n)	4 (3, 4.5)	4 (2.75, 6)	0.657
Parity (n)	2 (1.25, 2)	2 (2, 2.25)	0.674
Menstrual status			
Postmenopausal, n (%)	22 (91.7%)	20 (76.9%)	0.301
Premenopausal, n (%)	2 (8.3%)	6 (23.1%)	
HPV			
Positive, n (%)	23 (95.8%)	21 (80.8%)	0.229
Negative, n (%)	1 (4.2%)	5 (19.2%)	
SCC-Ag			
Positive, n (%)	14 (58.3%)	15 (57.7%)	0.963
Negative, n (%)	10 (41.7%)	11 (42.3%)	
Histopathology			
Squamous cell, n (%)	21 (87.5%)	20 (76.9%)	0.546
Other, n (%)	3 (12.5%)	6 (23.1%)	
FIGO stage			
IB, n (%)	10 (41.7%)	8 (30.8%)	0.423
IIA, n (%)	14 (58.3%)	18 (69.2%)	
Location			
Anterior lip of cervix, n (%)	13 (54.2%)	11 (42.3%)	0.185
Posterior lip of cervix, n (%)	8 (33.3%)	6 (23.1%)	
Both lips of cervix, n (%)	3 (12.5%)	9 (34.6%)	
Enhancement pattern			
Homogeneous, n (%)	8 (33.3%)	12 (46.2%)	0.355
Inhomogeneous, n (%)	16 (66.7%)	14 (53.8%)	
Maximum tumor depth/cervical radius			
<1/2, n (%)	1 (4.2%)	0 (0%)	0.480
≥1/2, n (%)	23 (95.8%)	26 (100%)	
Tumor volume (cm^3^)	3.63 (2.03, 11.09)	5.83 (2.34, 14.14)	0.332

Data are presented as mean ± standard deviation, median (lower quartile, upper quartile), or No. (%). LVSI, lymphovascular space invasion; HPV, human papilloma virus; SCC-Ag, squamous cell carcinoma antigen.

### 3.2 Intraobserver concordance of tumor volume and ADC histogram parameters

**Table 3** presents the intraobserver concordance in tumor volume and ADC histogram parameters. Among these parameters, the intraobserver concordance of ADC_min_ was not good (ICC = 0.453 [95% confidence interval (CI) 0.206-0.647], *P* < 0.001), and the average of the two measurements was used as the final analysis value. The intraobserver concordance of the remaining parameters was good or excellent (all ICC values > 0.60, *P* < 0.001), and the first measurements obtained by the reviewers working in consensus were used as the final values for further analysis.

**Table 3:** Intra-observer concordance of tumor volume and ADC histogram parameters.

Parameters	ICC (95%CI)	*P* value
Tumor volume	0.988 (0.972-0.994)	<0.001
ADC_min_	0.453 (0.206-0.647)	<0.001
ADC_max_	0.868 (0.780-0.923)	<0.001
ADC_range_	0.674 (0.490-0.800)	<0.001
ADC_mean_	0.978 (0.963-0.988)	<0.001
ADC_stdev_	0.776 (0.638-0.866)	<0.001
ADC_1_	0.879 (0.797-0.930)	<0.001
ADC_5_	0.979 (0.964-0.988)	<0.001
ADC_10_	0.988 (0.979-0.993)	<0.001
ADC_25_	0.995 (0.992-0.997)	<0.001
ADC_50_	0.993 (0.987-0.996)	<0.001
ADC_75_	0.970 (0.947-0.983)	<0.001
ADC_90_	0.893 (0.819-0.938)	<0.001
ADC_95_	0.859 (0.765-0.917)	<0.001
ADC_99_	0.813 (0.693-0.890)	<0.001
ADC_coefofvar_	0.874 (0.788-0.926)	<0.001
ADC_skewness_	0.734 (0.576-0.840)	<0.001
ADC_kurtosis_	0.698 (0.525-0.816)	<0.001
ADC_entropy_	0.839 (0.733-0.905)	<0.001

ADC, apparent diffusion coefficient; ICC, Intraclass correlation coefficient; CI, confidence interval.

### 3.3 ADC histogram parameters: LVSI-positive vs. LVSI-negative group

**Table 4** presents the results of the univariate analysis of ADC histogram parameters. The ADC_max_, ADC_range_, ADC_90_, ADC_95_, and ADC_99_ were significantly lower in the LVSI-positive group than in the LVSI-negative group (all *P*-values < 0.05); no significant differences were found in the remaining parameters between the groups (all *P*-values > 0.05).

**Table 4:** Comparisons of ADC histogram parameters between cervical cancer patients with and without LVSI.

Parameters	LVSI-positive group (*n*=24)	LVSI-negative group (*n*=26)	*P* value
ADC_min_ (×10^-3^ mm^2^/s)	0.58 (0.37, 0.65)	0.54 (0.45, 0.64)	0.861
ADC_max_ (×10^-3^ mm^2^/s)	1.72 ± 0.47	2.10 ± 0.41	0.004
ADC_range_ (×10^-3^ mm^2^/s)	1.16 (0.85, 1.50)	1.47 (1.33, 1.88)	0.003
ADC_mean_ (×10^-3^ mm^2/^s)	0.94 (0.87, 0.99)	0.97 (0.87, 1.10)	0.252
ADC_stdev_ (×10^-3^ mm^2^/s)	0.18 ± 0.07	0.22 ± 0.06	0.053
ADC_1_ (×10^-3^ mm^2/^s)	0.63 (0.57, 0.71)	0.66 (0.58, 0.75)	0.607
ADC_5_ (×10^-3^ mm^2^/s)	0.72 (0.63, 0.79)	0.74 (0.64, 0.82)	0.449
ADC_10_ (×10^-3^ mm^2/^s)	0.76 (0.69, 0.83)	0.77 (0.69, 0.88)	0.484
ADC_25_ (×10^-3^ mm^2/^s)	0.81 (0.76, 0.90)	0.83 (0.75, 0.96)	0.497
ADC_50_ (×10^-3^ mm^2^/s)	0.89 (0.84, 0.99)	0.92 (0.83, 1.06)	0.398
ADC_75_ (×10^-3^ mm^2^/s)	1.00 (0.94, 1.09)	1.04 (0.93, 1.21)	0.290
ADC_90_ (×10^-3^ mm^2^/s)	1.17 (1.03, 1.25)	1.25 (1.11, 1.39)	0.047
ADC_95_ (×10^-3^ mm^2^/s)	1.27 (1.12, 1.46)	1.46 (1.27, 1.53)	0.020
ADC_99_ (×10^-3^ mm^2^/s)	1.55 ± 0.40	1.82 ± 0.33	0.014
ADC_coefofvar_	0.17 (0.14, 0.27)	0.22 (0.16, 0.26)	0.197
ADC_skewness_	1.22 ± 0.88	1.67 ± 0.71	0.051
ADC_kurtosis_	1.81 (0.65, 4.85)	4.18 (1.84, 6.93)	0.087
ADC_entropy_	3.82 (3.58, 3.95)	3.74 (3.57, 3.92)	0.420

Data are presented as mean ± standard deviation, or median (lower quartile, upper quartile). LVSI, lymphovascular space invasion; ADC, apparent diffusion coefficient.

### 3.4 ROC analysis for predicting LVSI in stage IB-IIA cervical cancer

**Figure 2:**
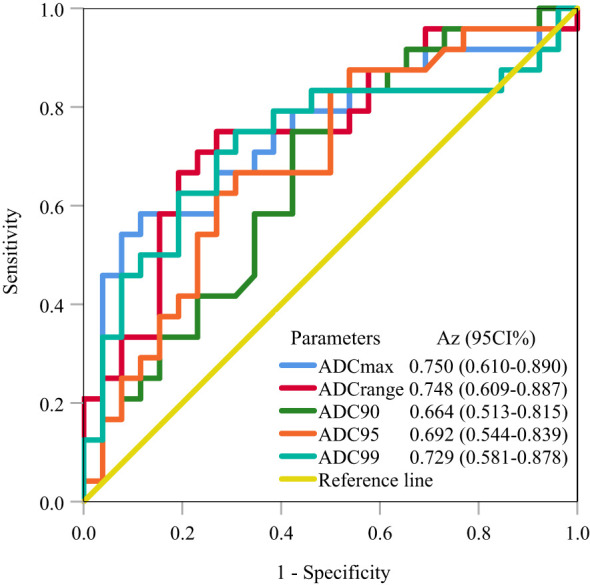
ROC analysis for predicting LVSI in stage IB-IIA cervical cancer. The ROC curves demonstrate that ADC_max_ achieved the largest A_z_ of 0.750, followed by ADC_range_ and ADC_99_ (A_z_ = 0.748 and 0.729, respectively). ROC, receiver operating characteristic; LVSI, lymphovascular space invasion; A_z_, area under the receiver operating characteristic curve.

ROC analysis for predicting LVSI in stage IB-IIA cervical cancer revealed that a cutoff ADC_max_ of 1.75×10^−3^ mm^2^/s achieved the largest A_z_ of 0.750, followed by a cutoff ADC_range_ of 1.36×10^−3^ mm^2^/s and ADC_99_ of 1.75×10^−3^ mm^2^/s (A_z_ = 0.748 and 0.729, respectively); the cutoff ADC_90_ and ADC_95_ achieved an A_z_ of <0.70 (**Table 5**; **Figure 2**).

**Table 5:** ROC analysis for predicting lymphovascular space invasion.

Parameters	Cut-off (×10^-3^ mm^2^/s)	A_z_ (95%CI)	Sensitivity(%)	Specificity(%)
ADC_max_	1.75	0.750 (0.610-0.890)	58.3	88.5
ADC_range_	1.36	0.748 (0.609-0.887)	75	73.1
ADC_90_	1.26	0.664 (0.513-0.815)	83.3	50
ADC_95_	1.34	0.692 (0.544-0.839)	66.7	69.2
ADC_99_	1.75	0.729 (0.581-0.878)	75	69.2

ROC, receiver operating characteristic; A_z_ area under receiver operating characteristic curve; CI, confidence interval.

## 4 Discussion

In this study, we evaluated the value of ADC histogram analysis based on the whole tumor volume in predicting LVSI in patients with stage IB-IIA cervical cancer. Our findings illustrated that ADC_max_, ADC_range_, ADC_90_, ADC_95_, and ADC_99_ obtained from the ADC histogram were lower in patients with LVSI than in patients without LVSI, and ADC_max_, ADC_range_, and ADC_99_ achieved moderate prediction values. These findings could be helpful for decisions on the extent of planned surgery and prognosis prediction in patients with stage IB-IIA cervical cancer, especially for women who require fertility.

As a quantitative parameter of DWI, the ADC value reflects the tumor microenvironment by detecting the diffusion of water molecules in the tumor and has been widely used for the diagnosis and prognosis prediction of cervical cancer (16, 17). However, most published studies have analyzed the average ADC value by drawing a region of interest (ROI) on a single layer of the tumor, which might lead to some useful information being missed. In this study, we used the VOI encompassing the entire tumor, which better reflects the heterogeneity of tumor than the ROI mentioned above. Meanwhile, ADC histogram analysis was applied to better assess the characteristics of cervical cancer. Compared with the traditional average ADC value, the ADC histogram analysis that we used can not only show the frequency distribution of ADC values of all voxels in the ROI but also avoid selection bias caused by the different placement of ROI.

In our study, ADC_max_, ADC_range_, ADC_90_, ADC_95_, and ADC_99_ were significantly different between cervical cancer patients with and without LVSI, and ADC_max_, ADC_range_ and ADC_99_ achieved moderate values for the prediction of LVSI. These results are not consistent with previous reports that demonstrated no statistical difference in ADC histogram between patients with and without LVSI (18, 19). The possible explanations are as follows: (1) the FIGO stages of the enrolled patients in the previous reports were different from those in our study and (2) VOIs of the tumor were drawn differently. In our study, hemorrhagic, necrotic, and cystic regions were included in the VOIs, whereas these areas were avoided in the previous report (19); and (3) ADC_max_, ADC_range_, ADC_95_, and ADC_99_, which were statistically different between the two groups in our study, were not analyzed in previous reports.

The diagnostic superiority of high percentile ADC values has also been demonstrated in tumors of various organs. Wang et al. (20) showed that ADC_90_ performed better than lower percentile ADC values in differentiating lymphoma from SCC and non-nasopharyngeal SCC. Guan et al. (21) reported that ADC_90_ is the strongest predictive parameter for discriminating cervical tumors from normal cervical tissues. Takahashi et al. (22) found that ADC_75_ had the best diagnostic ability in differentiating between uterine carcinosarcoma and endometrial carcinoma. In the current study, ADC_max_, ADC_range_, and ADC_99_ achieved superior performance in the prediction of LVSI compared with the other percentile ADC values, which may be related to the inclusion of hemorrhagic, necrotic, and cystic regions in the VOIs (23). These regions may have a variety of ADC values that may result in ADC_max_, ADC_range_, and ADC_99_ in tumors with LVSI differing more significantly from those in tumors without LVSI than ADC_mean_. Hence, ADC_max_, ADC_range_, and ADC_99_ demonstrated better prediction performance.

Unlike the high percentile ADC values that reflect intratumoral necrotic and cystic areas, the low-percentile ADC and ADC_min_ values reflect the densely packed solid components of the tumor (23). Although the presence of LVSI is a poor prognostic indicator of cervical cancer, it may not significantly alter the cellular density of the solid components of the tumor, and low-percentile ADC and ADC_min_ showed no significant differences between the two groups in this study. Additionally, the robustness of ADC_min_ is poor. It can be easily affected by extreme values resulting from noise, artifacts, or adjacent structure (24). In our study, the intraobserver concordance of ADC_min_ was also poor.

No significant differences were found in ADC_stdev_, ADC_coefofvar_, ADC_skewness_, ADC_kurtosis_, or ADC_entropy_, which are important parameters for evaluating the overall heterogeneity of tumors. This may be due to the inclusion of hemorrhagic, necrotic, and cystic regions in the VOIs, which may result in more complex ADC values and affect the spatial distribution of the ADC histograms. In addition, this may be associated with the small sample size of this study.

Our study has some limitations. First, it was a retrospective study with a small number of patients in a single center. A prospective multicenter, large-sample cohort study is needed to confirm our findings. Second, VOI was obtained by manual delineation along the margin of the tumor on diffusion-weighted images in our study, which was time-consuming. Finally, due to the small sample size in this study, we only evaluated the value of ADC histogram analysis in the preoperative prediction of patients with or without LVSI. Further studies should be designed to predict patients with focal or diffuse LVSI, which may be significant for prognosis prediction in patients with cervical cancer (25, 26).

In conclusion, our study demonstrates that whole-tumor ADC histogram analysis has potential value in preoperatively predicting LVSI in patients with stage IB-IIA cervical cancer. ADC_max_, ADC_range_, and ADC_99_ are promising prediction parameters.

## Data availability statement

The original contributions presented in the study are included in the article/supplementary material. Further inquiries can be directed to the corresponding authors.

## Ethics statement

The studies involving human participants were reviewed and approved by Affiliated Hospital of North Sichuan Medical College. Written informed consent for participation was not required for this study in accordance with the national legislation and the institutional requirements.

## Author contributions

J-MC: Methodology, Data curation, Formal analysis, Project administration, Writing – original draft preparation, Writing – review and editing. W-XL: Data curation, Project administration, Writing – review & editing. B-GT: Data curation, Formal analysis, Writing – review & editing. JP: Data curation, Formal analysis, Writing – review & editing. H-YZ: Conceptualization, Methodology, Project administration, Resources, Supervision, Writing – review and editing. T-WC: Conceptualization, Methodology, Resources, Supervision, Writing – review and editing. All authors contributed to the article and approved the submitted version.
